# *Salmonella enterica* exploits the auxin signaling pathway to overcome stomatal immunity

**DOI:** 10.1371/journal.ppat.1013662

**Published:** 2025-11-17

**Authors:** Brianna Fochs, Zachariah Jaramillo, Ho-Wen Yang, Jirachaya Yeemin, Maeli Melotto

**Affiliations:** 1 Department of Plant Sciences, University of California, Davis, California, United States of America; 2 Plant Biology Graduate Group, University of California, Davis, California, United States of America; 3 Horticulture and Agronomy Graduate Group, University of California, Davis, California, United States of America; The Ohio State University, UNITED STATES OF AMERICA

## Abstract

Stomata on the leaf surface can be entry ports for various microbes to gain access to the apoplast. Plants can prevent, or at least diminish, microbial internalization through the activation of pattern-triggered immunity (PTI) in guard cells, a phenomenon known as stomatal immunity. The human pathogen *Salmonella enterica* serovar Typhimurium strain 14028s (STm 14028s) can overcome PTI and reopen the stomata; however, the underlying mechanism has been elusive. Here, we provide evidence that biosynthesis of auxin from its precursor tryptophan in both the bacterium and the host plant is required for stomatal re-opening in response to STm 14028s, but not *Pseudomonas syringae* pv. *tomato* (*Pst*) strain DC3000. STm 14028s produces auxin on the leaf surface through indole pyruvate decarboxylase (*ipdC*) and it induces auxin biosynthesis in Arabidopsis likely through *YUC5*. The heterologous expression of STm 14028s *ipdC* in *Escherichia coli* serotype O157:H7 partially restores stomatal reopening, as this bacterium does not induce plant auxin biosynthesis. In addition, auxin signaling is required for STm 14028s-triggered stomatal re-opening evidenced by the lack of response in the *tir1-10, axr1-3*, and *aux1-7* mutants, or in the presence of auxin signaling inhibitors. Overall, our findings underline the unique role of auxin in the interaction between STm 14028s and Arabidopsis as mechanism to overcome stomatal immunity.

## Author summary

Currently, there is no technology available to eliminate human bacterial pathogens from internal tissues of edible leaves. However, bacteria can penetrate the leaf through stomatal pores on the surface. These pores can close and open in response to stimuli and be ports of entry for pathogens. Like several pathogenic phytobacteria, the non-typhoid *Salmonella enterica* can induce stomatal pore opening and gain access to the leaf extracellular space, making it crucial to understanding the underlying mechanisms of this phenomenon. In this study, we used omics analyses (exometabolomics and transcriptomics) and validation through genetic and pharmacological analyses to provide strong evidence that auxin biosynthesis and signaling in both the bacterium and the plant are required for the ability of *S. enterica* to fully open the stomatal pore. These findings show a novel mechanism for a bacterium to overcome stomatal immunity and highlight the specific interaction between *Salmonella* and Arabidopsis leaves.

## Introduction

Plants associate with microbial communities that inhabit their aerial parts especially the leaves, known as the phyllosphere [1]. Some of these microbes will persist on the leaf surface as epiphytes, while other microbes may also enter the leaf apoplast and become endophytes [2]. Transition to an endophytic lifestyle may occur through chemotaxis towards stomatal pores where bacteria, such as *Salmonella enterica* serovar Typhimurium (STm) 14028s and by *Pseudomonas syringae* pathovar *tomato* (*Pst*) DC3000, can enter the leaf tissue [3,4,5,6]. However, the plants respond to the presence of microbes through the recognition of microbe-associated molecular patterns (MAMPs) by pattern recognition receptors (PRR), triggering a signal cascade that closes the stomata and decreasing microbial entry [7]. This pattern-triggered immunity (PTI)-induced stomatal closure is known as stomatal immunity.

Although stomatal immunity restricts invasion by many microbes, some have a mechanism to counteract this response and subsequently reopen the stomata [reviewed by 5]. For instance, several species of phytopathogens deploy phytotoxins to reopen the stomata, such as coronatine (COR) production by *Pst* DC3000 that highjacks the jasmonoyl-*L*-isoleucine (JA-Ile) pathway [6] and fusicoccin production by the fungus *Fusicoccum amydali* that block abscisic acid (ABA)-induced closure of the stomatal pore [8]. *Xanthomonas* spp. also have several mechanisms of overcoming stomatal immunity such as deploying the type III effector (T3E) protein XopS [9] or use of the bacterial effector kinase XopC2 [10]. Both effectors activate jasmonate signaling to promote stomatal opening via interference with the JASMONATE ZIM-DOMAIN (JAZ) transcription repressors. While XopS binds to WRKY40 leading to repression of *JAZ8* expression in pepper [9], XopC2 specifically phosphorylate OSK1 thereby promoting the degradation of JAZ proteins in rice [10].

The human pathogen *S. enterica* is a unique microbe within the plant phyllosphere. This bacterium may come into contact with the leaf surface during the “farm-to-fork” food production chain likely through contaminated water in irrigation systems [11,12], proximity to cattle [13], and other sources [14,15,16,17], causing serious food-borne disease outbreaks, such as the ones reported by the US Centers for Disease Control and Prevention (https://www.cdc.gov/salmonella/). Once on the leaf surface, *S. enterica* moves towards nutrients leaked through the stomatal pore via chemotaxis [3]. Similar to what has been observed with phytopathogens, *S. enterica* triggers stomatal immunity [18] as the plant can recognize the *S. enterica*’s flagellin through the PRR-RLK FLAGELLIN SENSING 2 (FLS2) and causes the stomata to close [19]. Interestingly, *S. enterica* can reopen the PTI-closed stomata while another human pathogen, *Escherichia coli* serotype O157:H7, cannot [18]. *Salmonella enterica* can enter into the apoplast where it persists or multiply depending on the plant genotype [20].

Manipulation of hormone signaling pathways by effectors and phytotoxins is a common strategy for microbes to reopen PTI-closed stomata [21,6,7], making the leaf susceptible to microbial invasion (*i.e*., stomatal susceptibility). As *S. enterica* is capable of producing and secreting auxin through the indole-3-pyruvate (IPyA) pathway [22], we explored the function of auxin signaling in stomatal susceptibility to *S. enterica*. Auxin has important roles during plant-pathogen interactions [23], in addition to its well characterized functions in many physiological and biochemical processes of plant growth and development [24]. In plants, the biosynthesis of the most abundant biological form of auxin, indole-3-acetic acid (IAA), from its precursor tryptophan (Trp) takes place within the cytoplasm and involves various biosynthesis pathways [25]. Influx transporters, such as AUXIN RESISTANT 1 (AUX1), fine tune the concentration of auxin within the cell [26], which is perceived by the TRANSPORT INHIBITOR RESPONSE 1 (TIR1) protein [27]. Upon auxin perception, the repressor proteins AUXIN/INDOLE-3-ACETIC ACID (Aux/IAA) bind to TIR1, triggering the degradation of the Aux/IAA via the ubiquitination/26s proteosome system [28]. The degradation of the Aux/IAA repressors allows the auxin response factors (ARFs) to be released, thereby activating downstream auxin responsive genes [29]. Many of the auxin responsive genes belong to one of the following families: SAUR (Small auxin up RNA), Aux/IAA, and GH3 (Gretchen Hagen 3) [30,31].

In this study, we used plant and bacterium genetics, and functional genomics coupled with pharmacological and biochemical approaches to show that auxin biosynthesis in both Arabidopsis and STm 14028s are required for disarming stomatal immunity when they interact. We also demonstrate the uniqueness of this mechanism in the interaction of Arabidopsis with STm 14028s when compared to the phytopathogen *Pst* DC3000 and the human pathogen *Escherichia coli* serotype O157:H7. These findings underscore a distinct Arabidopsis-STm 14028s interaction and suggest this bacterium can adapt and use plants as hosts.

## Results

### Auxin signaling is required for stomata susceptibility to STm 14028s

**Fig 1:**
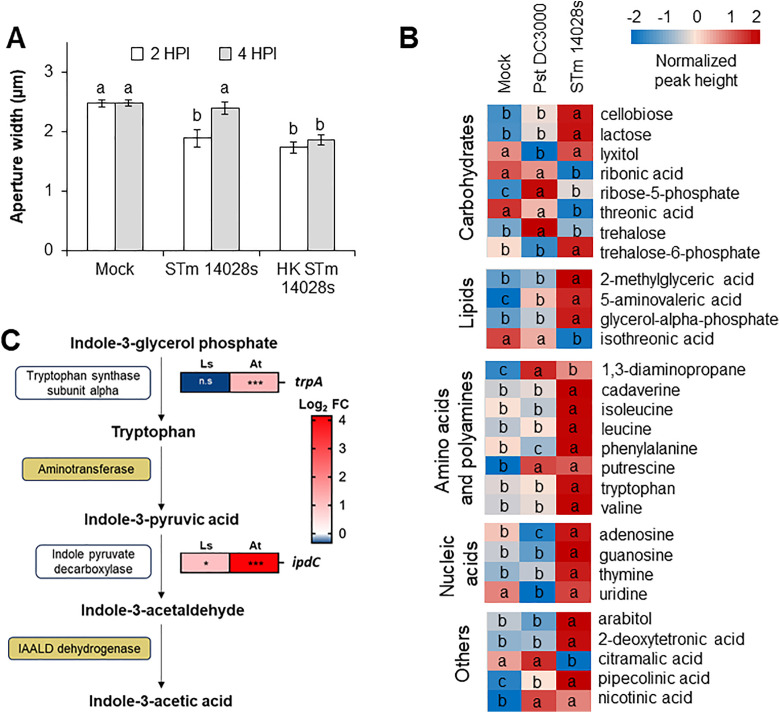
Auxin signaling is required for stomata susceptibility to *Salmonella enterica* (STm) 14028s. (A) Arabidopsis leaves were floated on either water as mock control or 1 x 10^8^ CFU.ml^-1^ bacterial inoculum containing STm 14028s or heat-killed (HK) STm 14028s. Measurements were taken at 2 and 4 hours post inoculation (HPI) and results are shown as the mean (n = 180-360) ± standard error (SE) from three independent experiments with three biological replicates each. Different letters on top of the bars represent statistically significant differences among the means determined via ANOVA and Scott-Knott (α = 0.05) (B) Heat maps of relative accumulation of DAMs in washes of leaves treated with mock, *Pst* DC3000, or STm 14028s inoculum. Peak heights of known differentially accumulated metabolites (DAMs) were normalized with Log_10_ transformation and auto scaling (mean-centered and divided by the standard deviation of each variable) functions of MetaboAnalyst software. Different letters on each box indicate significant statistical differences among the means, as calculated with ANOVA followed by Tukey’s test (α = 0.05). (C) A simplified auxin biosynthesis pathway regulated in STm 14028s when in contact with either Arabidopsis or lettuce leaves. On the right are the expression for the genes that encode the specific enzymes involved in the indicated metabolic step. Non-specific enzymes are in dark yellow boxes. Gene expression levels are shown as the average (n = 3) Log2 fold change (FC) [STm 14028s inoculum vs. 4 HPI on lettuce (Ls) or Arabidopsis (At)]. Significantly differential expression was considered for genes with a Benjanimi–Hochberg false discovery rate adjusted p-value, where * p < 0.05 and *** p < 0.001 [33].

Previously, we have shown that *Salmonella enterica* transiently closes the stomata of both lettuce and Arabidopsis at 2 hours post inoculation (HPI) [32,18]. We reasoned that this bacterium could secrete a compound to reopen stomata at 4 HPI, as it has been observed for other bacterial pathogens [6,9,10]. To test this hypothesis, we heat-killed (HK) the bacterium prior to conducting stomatal bioassays. Similar to the live STm 14028s control, the aperture width of stomata on leaves treated with HK STm 14028s was significantly smaller at 2 HPI than the mock-control. However, unlike the live bacterium treatment, the stomata did not reopen at 4 HPI when exposed to HK bacterium (Fig 1A).

Next, we conducted an exometabolomic analysis using leaf washes collected from mock-, STm 14028s-, or DC3000-treated samples at 4 HPI (S1A Fig) to identify secreted metabolites that could potentially open stomata. The list of all 438 metabolites and the 29 differentially accumulated metabolites (DAMs) detected with this analysis, and their relative accumulation in each sample are shown in S1 and S2 Datasets, respectively. Interestingly, PCA and hierarchical clustering analysis showed that STm 14028s-treated samples have a distinct metabolome profile compared to DC3000- and mock-treated samples, and the latter two have largely overlapping metabolome profiles (S1B,C Fig). We also observed that most known metabolites (21 out of 29), classified as carbohydrates, lipids, amino acids and polyamines, nucleic acids, and others, were significantly hyperaccumulated in the STm 14028s-treated samples when compared to other treatments (Fig 1B).

To further select specific metabolites that could contribute the most for the STm 14028s-triggered stomatal opening, we searched our previous transcriptomic dataset [33] for STm 14028s genes that were significantly upregulated after 4 hours of exposure to both Col-0 and lettuce leaves when compared to the inoculum alone. Among the 34 significantly upregulated STm 14028s genes (S3 Dataset), we found *ipdC* (gene ID MC58_02340) that encodes indole pyruvate decarboxylase, the rate-limiting enzyme for auxin biosynthesis in *Salmonella* [22]. Furthermore, the biosynthesis gene for the auxin precursor tryptophan, *trpA* [34] (gene ID MC58_07735), was also significantly upregulated in STm 14028s exposed to Arabidopsis leaves (S3 Dataset and Fig 1C). The upregulation of *ipdC* and *trpA* and the significant increase of tryptophan in the leaf exudate specifically inoculated with STm 14028s (Fig 1B) led us to hypothesize that auxin could be a major player in stomatal opening in response to this bacterium.

**Fig 2:**
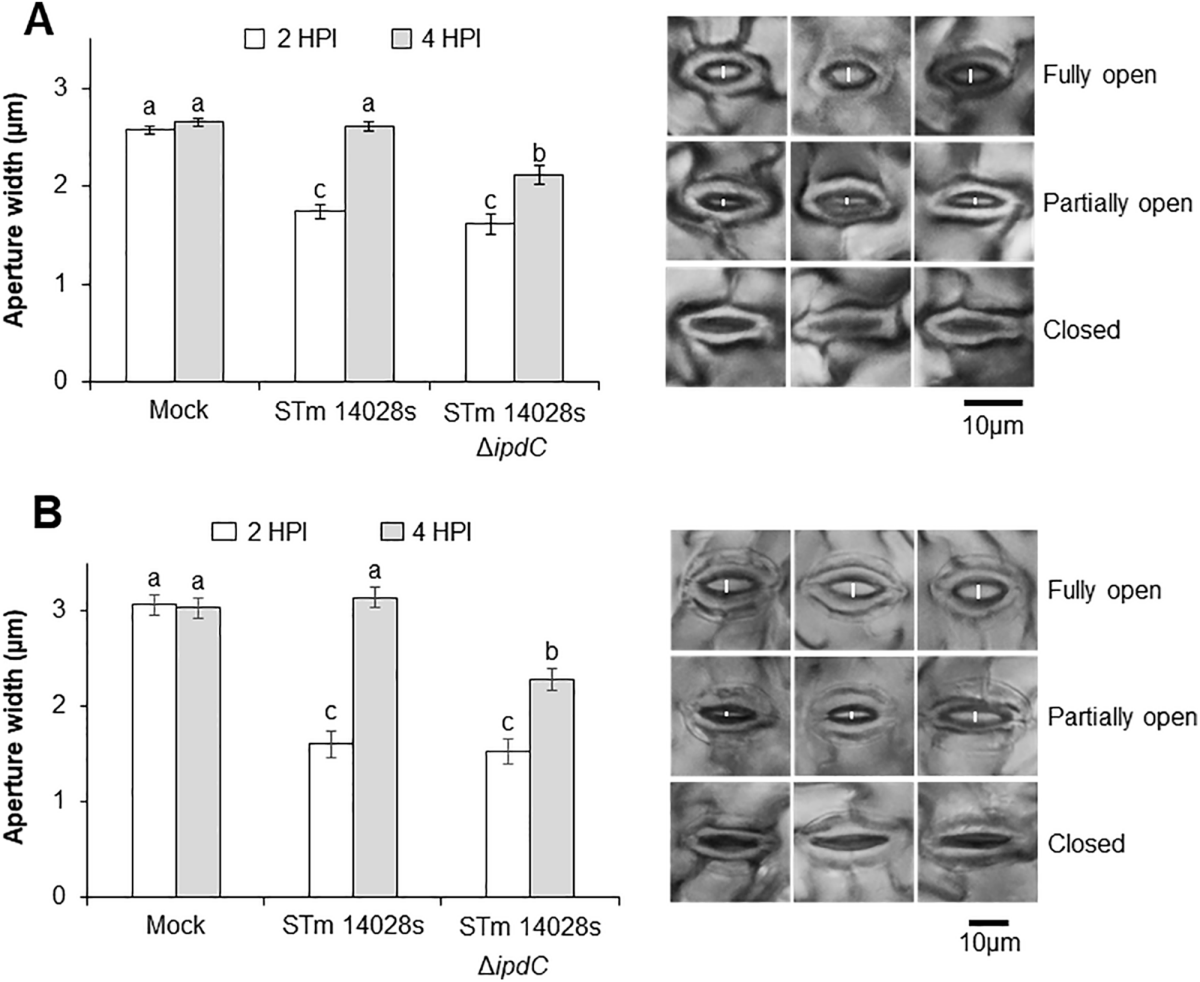
Bacterium auxin biosynthesis contributes to stomata susceptibility to *Salmonella enterica* (STm) 14028s. Stomatal aperture width in Col-0 (A) and lettuce (B) leaves floated on either mock (water), STM 14028s, or STm 14028s ∆*ipdC* inoculum at 1 x 10^8^ CFU.ml^-1^. Stomatal aperture width was measured at 2 and 4 hours post inoculation (HPI) and results are shown as the mean (n = 180-360) ± standard error (SE) of stomatal aperture width from three independent experiments with three biological replicates each. Different letters on top of the bars represent statistically significant differences among the means determined via ANOVA, followed by a Scott-Knott test (α = 0.05). Micrographs on the right represent stomata of Col-0 (A) and lettuce (B) with different pore aperture widths (open, partial, and closed). The white vertical line on the pore depicts the measurement used to create the graphs.

To assess whether bacterium-derived auxin has a prominent role in stomatal reopening, we obtained an STm 14028s strain lacking the *ipdC* gene (STm 14028s ∆*ipdC*). Interestingly, this mutant partially reopened Col-0 stomata at 4 HPI (Fig 2A). Thus, we suspected that plant-produced auxin could also contribute to stomatal re-opening. Next, we assessed the ability of STm 14028s ∆*ipdC* to re-open stomata of lettuce, and observed that it did so significantly, but partially (Fig 2B) as observed for Col-0 (Fig 2A). These findings suggest that bacterium- and plant-derived auxin signaling contribute to stomatal susceptibility to this human pathogen in at least two plant species.

### Bacterium-derived auxin contributes to stomatal reopening

**Fig 3:**
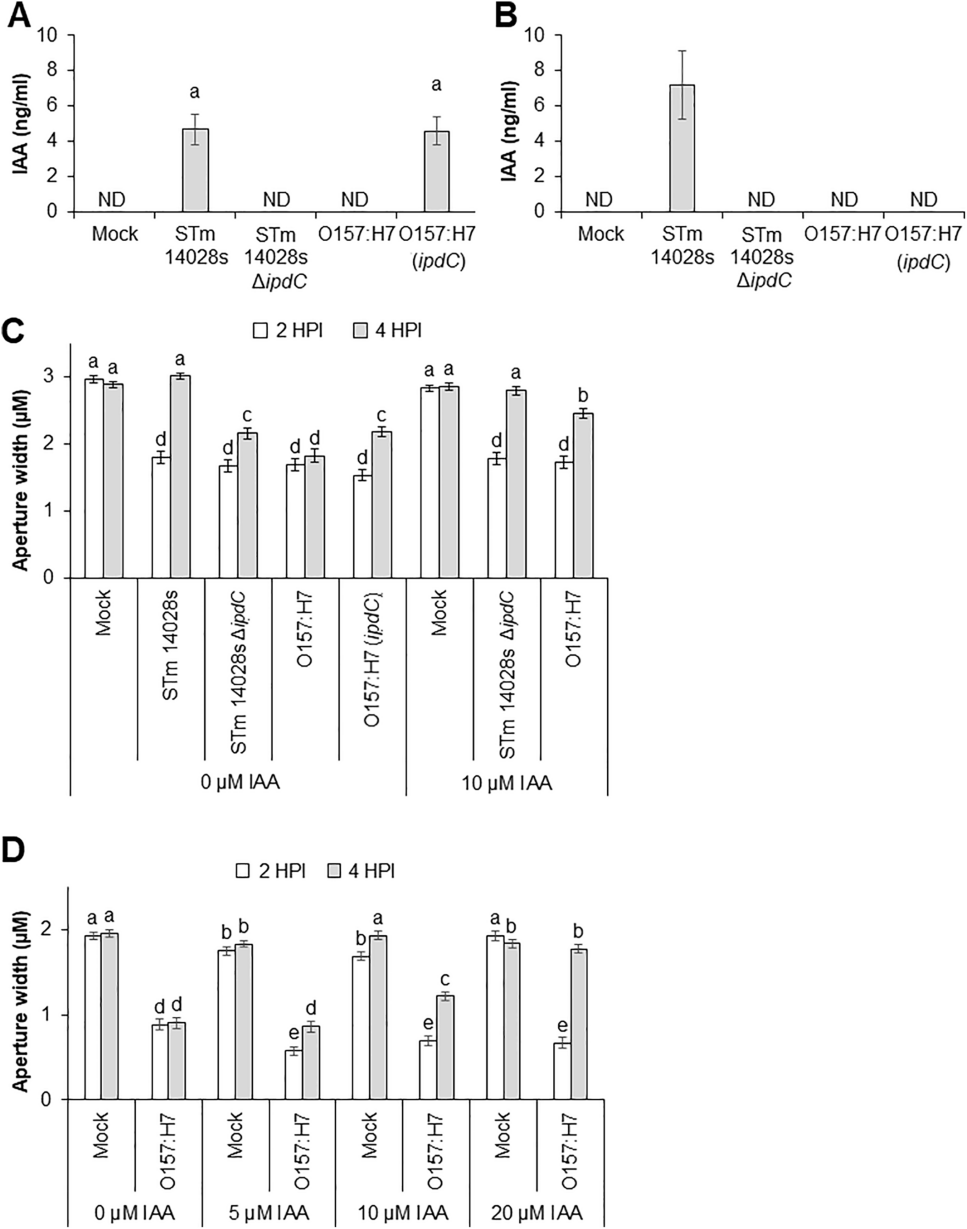
Bacterium-derived auxin contributes to stomatal reopening. (A) IAA concentration produced by STm 14028s, STm 14028s ∆*ipdC*, O157:H7, O157:H7 (*ipdC*), and *Pst* DC3000 in culture supplemented with tryptophan (1 mM) and grown for 72 hours. (B) IAA concentration in the leaf phyllosphere at 4 HPI with mock or bacterial (5 x 10^8^ CFU.ml^-1^) inoculum. (C, D) Four-week-old Arabidopsis leaves were inoculated with either mock or bacterial inoculum of STm 14028s, STm 14028s ∆*ipdC*, O157:H7, O157:H7 (*ipdC*). Some inocula were supplemented with 5, 10, or 20 µM IAA for the duration of the experiment. For all graphs, the results are shown as the average of three independent experiments ± standard error (SE), in which three biological replicates were included. Different letters on top of the bars represent significant statistical differences among the means calculated by ANOVA followed by Scott-Knot test (α = 0.05). ND = not detected, where the detection limit of the analysis was 0.6 ng/ml.

To further confirm that bacterium-derived auxin is important for stomatal susceptibility, we used a heterologous system to deliver auxin. The wild type *E. coli* O157:H7 cannot overcome stomatal immunity [6] or produce auxin via IpdC [22]. Thus, we expressed the encoding gene in *E. coli* O157:H7 to create a gain-of-function auxin biosynthesis strain, O157:H7 (*ipdC*) (S2 Fig). First the capacity of this strain to produce IAA was confirmed. When supplemented with tryptophan, both O157:H7 (*ipdC*) and STm 14028s secreted IAA to the medium at similar level (Fig 3A). As expected, no IAA was produced by STm 14028s ∆*ipdC* or O157:H7 in this assay (Fig 3A).

Next, we assessed IAA accumulation in the phyllosphere of leaves challenged with all bacterial strains at 4 HPI. We were able to detect IAA secreted to the phyllosphere of STm 14028s-treated leaves but not to the phyllosphere of mock-, STm 14028s ∆*ipdC-,* O157:H7-, or O157:H7 (*ipdC*)-treated leaves (Fig 3B). The lack of IAA detection in exudates of O157:H7 (*ipdC*)-treated leaves suggests that either the IAA level was induced at a level below the 0.6 ng/ml detection limit of the assay, or the plant fails to produce IAA during O157:H7-Arabidopsis interaction.

Stomatal bioassays on Arabidopsis leaves showed that O157:H7 (*ipdC*) can induce the opening of stomata to a significantly greater extent than that of the wildtype O157:H7 (Fig 3C). However, this heterologous expression of *ipdC* did not give the strain the capability to reopen stomatal apertures to the same level as the wildtype STm 14028s. Interestingly, O157:H7 (*ipdC*) and STm 14028s ∆*ipdC* induced comparable increase of stomatal aperture width at 4 HPI (Fig 3C). These results suggest that the partial stomatal aperture re-opening is due to the reduced amount of auxin in the phyllosphere (Fig 3B). To further support this hypothesis, we supplemented STm 14028s ∆*ipdC* and wildtype O157:H7 inoculum with exogenous auxin to compensate for their auxin biosynthesis impairment. The addition of 10 µM IAA to STm 14028s ∆*ipdC* resulted in stomatal re-opening to a similar extent to the wildtype STm 14028s. IAA also promoted O157:H7-induced stomatal opening significantly when compared to the O157:H7 (*ipdC*) strain; however, the average aperture opening was still significantly lower than the 10 µM IAA mock control (Fig 3C). Thus, we performed an IAA dose response experiment to test whether supplementation with increased IAA concentration would allow O157:H7 to fully open stomata. A gradient of stomatal re-opening in response to O157:H7 as the IAA concentration increased was observed, and 20 µM IAA was sufficient to complement stomatal re-opening to the full extent (Fig 3D).

### Upregulation of plant auxin biosynthesis contributes to stomatal reopening by STm 14028s

To start analyzing the plant auxin signaling pathways that could contribute to stomatal susceptibility to STm 14028s, we conducted a detailed search into our previous dual transcriptomic analysis of STm 14028s inoculated Arabidopsis [33]. This analysis revealed that several Arabidopsis genes involved in the auxin signaling pathway were modulated by STm 14028s, including the regulation of genes encoding many enzymes for Trp biosynthesis and genes involved in auxin biosynthesis (*YUC5*), transport (*AUX1, LAX1, LAX2, LAX3*), and signaling (including *TIR1* and members of the *AFB*, *ARF*, and *IAA* gene families) (S3 Fig, S4 Dataset).

The transcriptomic data revealed the differential regulation of genes involved in two branches of auxin biosynthesis, the indole-3-acetonitrile pathway (IAN) and the indole-3-pyruvic acid pathway (IPA). We found that many of the genes in the IAN pathway, such as *CYP79B2, CYP71A12* and *NIT2,* were significantly upregulated (S3 Fig). In contrast, many of the genes in the IPA pathway were downregulated such as *TAA1, YUC8* and *YUC9*, while the expression of *YUC6* was not regulated and the expression of *YUC5* was significantly upregulated (S3 Fig).

**Fig 4:**
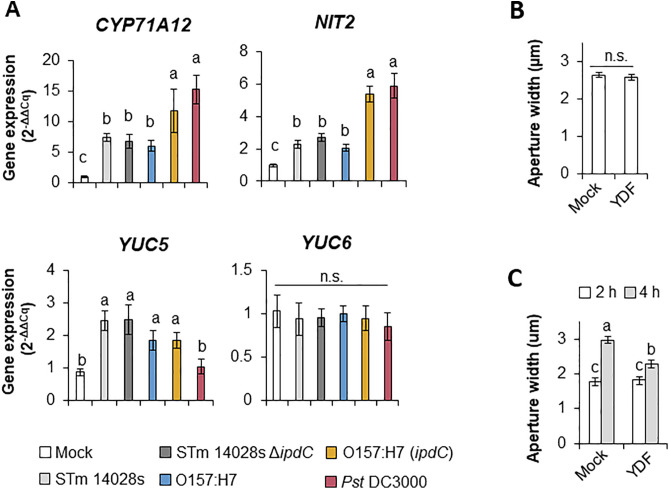
Arabidopsis auxin biosynthesis contributes to STm 14028s-induced stomatal reopening. (A) RT-qPCR analysis of the genes *CYP71A12*, *NIT2*, *YUC5*, and *YUC6*, which are involved in Arabidopsis auxin biosynthesis. Gene expression was assessed at 4 hours post inoculation with either a mock solution (0.004% Silwet) or a 1 x 10^8^ CFU.ml^-1^ bacterial inoculum of *Salmonella enterica* 14028s (STm 14028s), STm 14028s ∆*ipdC*, *Escherichia coli* O157:H7 (O157:H7), O157:H7 (*ipdC*), and *Pseudomonas syringae* DC3000 (*Pst* DC3000). All gene expression levels are relative to *PP2AA3* (At1G13320) based on ΔCq values and further normalized to the mock treatment using the ΔΔCq method. Data points are the mean of four independent experiments (n = 4) ± standard error (SE). Different letters above the bars indicate statistical significance calculated by ANOVA and Scott-Knot test (α = 0.05). (B) Four-week-old Arabidopsis leaves were pretreated with either water (mock) or 250 µM of Yucasin-DF (YDF) for 30 minutes and stomatal aperture was measured 2 hours post pre-treatment. The data points are the mean of stomatal aperture width (n = 60) ± SE. Significance was determined by a two-tailed Student’s t-test (p ≤ 0.05). (C) Mock- or YDF-treated leaves were floated on STm 14028s inoculum (1 x 10^8^ CFU.ml^-1^). The data points are the mean of stomatal aperture width (n = 60) ± SE measured at 2 and 4 hours post incubation. Significance was calculated between adjacent data points by ANOVA and followed by a Scott-Knot test (α = 0.05). Different letters above the bars indicate significant statistical differences. n.s. = not significant.

The expression level of the aforementioned auxin biosynthesis genes was assessed through RT-qPCR assays to confirm the transcriptomic data. Furthermore, to determine the specificity of the response we used the various bacterial strains for this assay, including the phytopathogen Pst DC3000 that also re-opens stomata through the JA-Ile pathway [6]. *CYP71A12* and *NIT2* were significantly upregulated by all bacterial strains when compared to the mock control (Fig 4A). Interestingly, *YUC5* was significantly upregulated only by human pathogenic strains, whereas *YUC6* was not modulated by any bacteria used in this assay (Fig 4A).

To support the role of YUC in STm 14028s-induced stomatal opening, we used yucasin-DF (YDF) that competitively inhibits YUC enzymes [35] to specifically block auxin biosynthesis in the plant prior to inoculation with STm 14028s. Pre-treatment with YDF had no effect on stomatal aperture in non-inoculated leaves (Fig 4B), whereas it did disrupt the reopening of stomata by STm 14028s as the apertures are significantly smaller when compared to that of the control samples (Fig 4C). However, STm 14028s partially re-opened the stomata of YDF-pretreated leaves, further supporting that bacterium-derived auxin also contributes to the phenotype (Figs 2, 3).

Altogether, these findings suggest that STm 14028s modulates and requires Arabidopsis auxin biosynthesis via the Trp and YUC5 to overcome stomatal defense.

### Plant auxin signaling is required for stomatal susceptibility to STm 14028s

**Fig 5:**
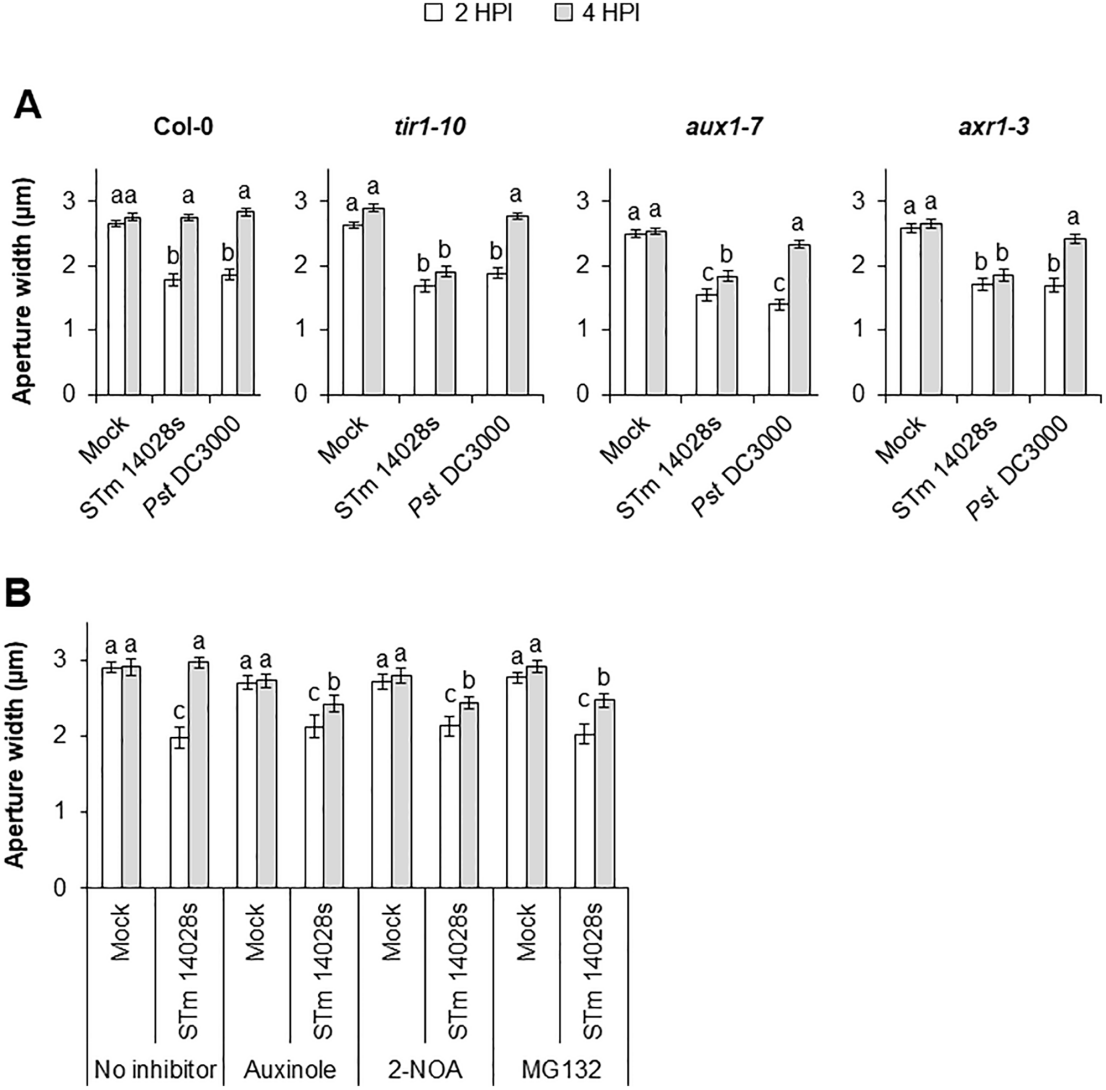
Impairment of the Arabidopsis auxin signaling pathway disrupts *Salmonella enterica’s* capability to reopen the stomata. Stomatal aperture width of 4- to 5-week-old Arabidopsis leaves at 2 HPI and 4 HPI. (A) Col-0, *tir1-10, aux1-7* and *axr1-3* leaves were floated on either mock (water) or a bacterial inoculum of either STm 14028s or *Pst* DC3000 (1 x 10^8^ CFU.ml^-1^). Results are shown as the mean of three independent experiments (n = 180) ± standard error (SE). (B) Leaves of Col-0 were pretreated with water (no inhibitor), auxinole (100 µM), 2-naphthoxyacetic acid (2-NOA) (100 µM), or MG132 (100 µM) for 30 minutes prior to float-inoculation with either mock or STm 14028s (1 x 10^8^ CFU.ml^-1^). Results are shown as the mean of three independent leaves (n = 60) ± standard error (SE). Different letters represent statistical differences among the means, as calculated by a two-way ANOVA and followed by a Scott-Knot test (α = 0.05).

To provide further evidence that the Arabidopsis auxin pathway is involved in STm 14028s-triggered stomatal re-opening, we assessed the stomatal movement in leaves of three selected auxin signaling mutants, *tir1-10*, *aux1-7,* and *axr1-3* [S4 Fig; 27,36,26]. We found that impaired auxin signaling pathway does not disrupt PTI response as the stomata of these mutants still close in both STm 14028s- and *Pst* DC3000-inoculated leaves at 2 HPI (Fig 5A). However, STm 14028s could not re-open any of the auxin mutant stomata to the level of the wildtype stomata at 4 HPI (Fig 5A). Interestingly, *Pst* DC3000 still reopens the stomata of *axr1-3, aux1-7,* and *tir1-10* to an aperture width similar to that of Col-0 (Fig 5A).

To rule out the possibility that the mutations of *axr1-3, aux1-7*, and *tir1-10* lead to abnormal stomatal development that would influence our results, we quantified several stomatal traits in these mutants. We did not find any significant differences between the mutant genotypes when compared to the wildtype Col-0 for stomatal aperture width (A), total width of the stomatal complex (W), stomatal complex length (L), guard cell pair width (W-A), total stomatal complex area (W*L) and stomatal density on the abaxial surface of the leaf (S5 Fig). These findings confirm that stomata of plant mutants are not morphologically distinct from Col-0, and the results of the stomatal bioassays are due to the modulation of the auxin pathway by STm 14028s to reopen the stomata.

To further confirm the importance of a functional plant auxin signaling in stomatal susceptibility to STm 14028s, we used a pharmacological approach by using the inhibitors auxinole, MG-132, and 2-naphthoxyacetic acid (2-NOA) prior to stomatal bioassays. Auxinole directly binds to and inhibits SCF^TIR1^ [37], MG-132 is an inhibitor of the 26S proteasome, and 2-NOA is an auxin transport inhibitor that blocks the uptake of auxin into plant cells by AUX1 [38]. Pre-treatment with these inhibitors had no effect on stomatal movement as the aperture width did not change in comparison to non-treated leaves (Fig 5B). However, these inhibitors did disrupt the ability of STm 14028s to fully reopen the stomata, as the mean aperture in inhibitor pre-treated leaves was significantly smaller than the aperture of non-treated leaves at 4 HPI (Fig 5B).

## Discussion

**Fig 6:**
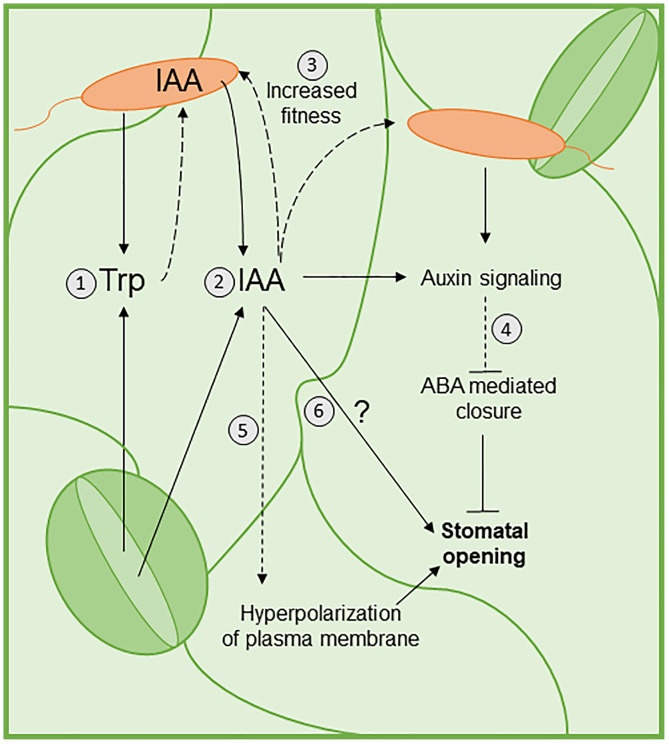
A proposed model for the role of auxin in plant-*Salmonella* interaction. (*1*) Both STm 14028s (*orange*) and the plant produce tryptophan (Trp) which is released onto the leaf surface [33]. (*2*) The Trp on the leaf surface can be utilized by STm 14028s to produce higher concentrations of IAA. The plant also produces IAA in response to the bacterium. (*3*) The high IAA concentrations on the leaf surface can increase the fitness of the bacteria [22]. (*4*) Both STm 14028s and the concentration of auxin activate the plant auxin signaling pathway, which could inhibit ABA-mediated closure and thus allow the stomata to reopen [41,33,42]. (*5*) Increased IAA could also allow for stomatal reopening, potentially through the hyperpolarization of the K^+^ channels of the plasma membrane of the stomata [43]. (*6*) IAA could directly control stomatal movement through an unknown mechanism. Solid lines indicate support from our results or known pathways, while dashed lines indicate hypotheses based on the literature.

Plants can recognize *Salmonella* flagellin, thereby inducing PTI responses such as stomatal immunity [19,39,18]. However, the ability of this human pathogen to reopen stomata of several leafy vegetables [40] may have serious consequences for the safety of freshly consumed leafy greens. Currently, there is no technology to eliminate endophytic populations of human pathogens, making it crucial to elucidate *Salmonella*’s counter defense mechanisms and be able to mitigate the risk of foodborne illnesses. Here, we show that auxin biosynthesis in both the plant and the bacterium is required for full reopening of stomata when they come into contact and offer a model for the underlying mechanism (Fig 6).

In Arabidopsis, auxin biosynthesis occurs via a well characterized Trp-dependent pathway and in a less understood Trp-independent pathway [24,44]. Our observations that Trp biosynthesis genes have increased expression in both STm 14028s (Fig 1C) and Arabidopsis (S3 Fig), accompanied by an elevated level of Trp in the phyllosphere of STm 14028s-treated leaves (Fig 1B), indicate that the Trp-dependent pathway for auxin biosynthesis might have a predominant role in STm 14028s-induced stomatal opening. Trp itself might induce stomatal closure as previous studies showed that some, but not all, Trp biosynthetic mutants (such as *tsb1-1* and *trp3-1*) have greater stomatal aperture than the wild type and exogenous application to Trp rescues the wild type phenotype [45]. These authors also reported that the *tsb1-1* stomata phenotype was not related to auxin [45]. While the role of Trp in stomatal movement is unclear, the elevated Trp in STm 14028s-Arabidopsis interaction might increase the bacterium’s fitness in the phyllosphere. For instance, Trp genes have increased expression in *Salmonella* biofilms [46], which is an important mechanism for *Salmonella* attachment to leaves [47,3,48]. Furthermore, elevated Trp levels could lead to higher auxin biosynthesis and secretion, which in turn opens the stomatal pore (Fig 6).

Many strains of bacteria can produce IAA [49,50,51,52,53], including *Salmonella enterica* [Fig 3, 22]. The human body is also capable of producing IAA through *Interleukin-4-Induced gene 1* (*IL4I1*) [54,55] and IAA is present in the gut microbiome [56], an environment in which *Salmonella* is adapted to live and interact with the intestinal microbiota [57]. Interestingly, it has been reported that STm 14028s ∆*ipdC* is less virulent on the mouse gut, indicating the importance of IAA production in STm 14028s’s survival [22]. The secretion of biologically relevant concentrations of auxin or exogenous application of IAA to the bacterial inoculum are sufficient for bacterium-triggered reopening of stomata at 4 HPI (Fig 3). Furthermore, auxin perception and signaling by the plant are required to express this phenotype (Fig 5). Thus, it is not surprising that STm 14028s-derived IAA contributes to the bacterium’s fitness in the phyllosphere and is a strategy to overcome stomatal immunity (Fig 3). Notably, the auxin action on the physiology of both the plant and bacterium during their interaction has also been observed in the Arabidopsis-DC3000 system, albeit at a later stage of infection [58].

Although the impact of auxin on stomatal movement has been demonstrated, this process is not as well understood as for other phytohormones, such as ABA, salicylic acid, and jasmonates [59,60,61,62,63,64]. Nonetheless, exogenous application of auxin can cause stomatal opening [65,66,67,42] and blocking endogenous auxin through the auxin antagonist α-(phenyl ethyl-2-one)-IAA reduces stomatal aperture size [68]. In addition, evidence suggests that auxin may be an antagonist to ABA-induced stomatal closure [41,68,42], and ABA signaling is required for stomatal immunity [6]. For instance, mutants of auxin response factors, *arf2* in both Arabidopsis and tomato and *arf4* in tomato have elevated endogenous ABA levels and decreased stomatal conductance when compared to the wild type plants [69,70,71]. These results suggest an antagonistic relationship between auxin signaling and ABA content, resulting in alteration in the stomatal aperture. Furthermore, exogenous IAA can block ABA-induced stomatal closure [41,42]. Our transcriptome data also show that members of the *PP2C* family and *ABI1*, negative regulators of ABA signaling [72,73], are significantly upregulated, whereas many ABA biosynthesis genes are down-regulation in response to STm 14028s (S4 Dataset). Thus, it is possible that ABA signaling is suppressed due to elevated auxin accumulation in the phyllosphere in the presence of STm 14028s (Fig 3), which is associated with the activation of biosynthesis gene expression (*TSB1/2/4* and *CUP71A12, NIT2, and YUC5*) (Figs S3 and 4) and Trp accumulation (Fig 1B).

The impairment in stomatal reopening in the mutant plants *aux1–7*, *axr1–3,* and *tir1–10* (Fig 5), suggests that a functional auxin signaling pathway is required for stomatal susceptibility to STm 14028s. While the role of AXR1 and TIR1 on stomatal movement is poorly understood, previous studies have linked AUX1 to stomatal opening, as inhibition of auxin transport by 2-NOA disrupts the ability of IAA to prevent ABA-induced stomatal closure [41], further supporting that auxin blocks ABA-mediated stomatal closure [42]. Although, we show evidence that auxin antagonizes ABA during Arabidopsis-STm 14028s interaction, additional mechanisms underlying this process cannot be discarded. For example, IAA can hyperpolarize the plasma membrane, activating inward K^+^ channels to cause stomatal opening [43] and it could be a subject for future investigations.

## Materials and methods

### Plant material and growth conditions

*Arabidopsis thaliana* ecotype Columbia (Col-0, CS60000), and its derived mutant lines *aux1–7* (CS3074), *axr1–3* (CS3075), and *tir1–10* (SALK_090445C) were genotyped for the alleles of interest at the corresponding locus *AUX1* (At2g38120), *AXR1* (At1g05180), and *TIR1* (At3g62980), according to the procedure outlined at The Arabidopsis Information Resource database (https://www.arabidopsis.org/). Briefly, alleles were identified by PCR (Gotaq, Promega, Madison, WI, USA) in accordance with the manufacturer’s guidelines with various primers (S1 Table) to confirm homozygosity of the mutant line. The *tir1–10* allele and the equivalent wild type were detected with allele-specific primer sets. To detect the *aux1–7* and *axr1–3* alleles, only one primer set was used for each gene. Mutant alleles were differentiated from wildtype alleles by cutting PCR products with a restriction enzyme. For *aux1–7,* DpnII (New England BiolabsNEB, Ipswich, MA, USA) was used, which cuts the mutant allele three times and the wildtype allele twice. AccI (NEB) was used for *axr1–3,* which only cut the wildtype allele.

Arabidopsis seeds were suspended in 0.1% agarose and kept at 4 °C in the dark for 24–48 hours to ensure synchronous germination. Prior to sowing seeds, soil (Sun Gro Sunshine #1 Professional Growing Mix, Sun Gro Horticulture, Agawam MA, USA) was put into pots and soaked with an aqueous solution of 1 g.L^-1^ Gnatrol (Valent, Canada) overnight. A thin layer of fine vermiculite (Thermo-O-Rock, New Eagle, PA, USA) was placed on top of the soil, and pots were covered with mesh. Seed suspension was pipetted onto soil, the pots were placed on trays that were covered with plastic domes and placed in a growth chamber with the following conditions: 22 °C, 65 ± 5% relative humidity (RH), 12-hour photoperiod (100 µmol.m^-2^.s^-1^). Once germination started (approximately 2–3 days), domes were removed for the remainder of the experiment. Plants were watered by adding tap water to the trays as needed until the end of experimentation.

Seeds of the lettuce (*Lactuca sativa* L.) cultivar Salinas were sown on water-soaked germination paper inside of covered square Petri dishes and incubated for two days at 22 °C on a 12-hour photoperiod. Germinated seeds were transferred to pots containing soil (Sun Gro Sunshine #1 Professional Growing Mix) and grown under a light intensity of 240 ± 10 µmol.m^-2^.sec^-1^ with a 12-hour photoperiod, with day and night conditions being 19 ± 2 °C and 74 ± 4% RH and 18 ± 1 °C and 92 ± 2% RH, respectively. Trays were covered with plastic domes until plants were used in experiments and water was added to the trays as needed.

### Bacterial strains and inoculum preparation

*Salmonella enterica* subspecies *enterica* serovar Typhimurium strain 14028s (STm 14028s), STm 14028s *ipdC* null mutant (STm 14028s ∆*ipdC*, gift from Michael McClelland), *Escherichia coli* serotype O157:H7 (O157:H7, gift from Vanessa Sperandio), gain of function mutant *ipdC* (O157:H7 (*ipdC*)), and *Pseudomonas syringae* pathovar *tomato* strain DC3000 (*Pst* DC3000, gift from Sheng Yang He) were grown from single colonies in Low Salt Luria Bertani (LBLS) medium (10 g.L^-1^ tryptone, 5 g.L^-1^ yeast extract, 5 g.L^-1^ NaCl, pH 7.0) supplemented with the appropriate antibiotic as follow: none (STm 14028s), 20 μg.ml^-1^ chloramphenicol (STm 14028s ∆*ipdC*), 50 μg.ml^-1^ streptomycin (O157:H7), 50 μg.ml^-1^ streptomycin and 50 μg.ml^-1^ kanamycin (O157:H7 (*ipdC*)), and 100 μg.ml^-1^ rifampicin (*Pst* DC3000). Cultures were grown overnight in 28 °C to an OD_600_ of 0.8 – 1.0. Bacterial cells were collected via centrifugation at 2600 rpm at room temperature and resuspended in sterile MilliQ water (Barnstead NANOpure DIamond Ultrapure Water System, Lake Balbo, CA, USA) to the desired concentration.

### Stomatal bioassays

The night prior to inoculations, 4- to 5-week-old Arabidopsis or 3-week-old lettuce were covered with plastic domes that had been sprayed in water to ensure a humid enough environment for stomata to be open prior to the experiment. For all experiments, three leaves from the middle layer of the plant were used and the experiments were conducted between three and seven hours after first light.

When pre-treating leaves with inhibitors, detached leaves were floated on their abaxial side on either water, 250 µM yucasin difluorinated [YDF; a gift from Ken-ichiro Hayashi, 35], 100 µM auxinole [a gift from Ken-ichiro Hayashi, 35], 100 µM MG132 (Cayman Chemicals, Ann Arbor, Michigan, USA), or 100 µM 2-naphthoxyacetic acid (2-NOA) (Sigma-Aldrich, MO, USA) for 30 minutes and then blotted prior to float-inoculation. Subsequently, leaves were floated in small petri dishes (60x15mm) with approximately 12.5 mL of either water or 1 x 10^8^ CFU.ml^-1^ bacterial inoculum. Experiments with exogenous auxin had 5, 10, or 20 µM IAA (Research Products International, Mt. Prospect, IL, USA) added to water (mock) or the inoculum. To heat kill STm 14028s, the bacterial inoculum was placed at 100 °C for 10 minutes and the inoculum was plated on LSLB medium to ensure all cells were dead.

All stomata were imaged using a Nikon Eclipse Ni-U upright microscope with 20x magnification, long distance objective, and without cover slip. Images were analyzed with Nikon NIS Elements software (Version 4.13). Data points are shown as the mean of three independent experiments (n = 60–180) ± standard error (SE). Statistically significant differences were calculated by two-way ANOVA followed by a comparison of means using the Scott-Knott test (α = 0.05).

To ensure the auxin mutant plants had a similar stomatal morphology to that of the wildtype, we measured the stomatal aperture (A), stomatal width (W), and stomatal length (L) of the same stomata (n = 100). Measurements were used to calculate the guard cell (GC) pair width (W-A) and the area of the stomatal complex (W x L). Stomatal density was calculated by counting the number of stomata in a 1 mm^2^ area on different leaves (n = 20). Data points represent the means ± SE. Statistical difference was determined by comparing mutants to the wildtype using a two-tailed Student’s t-test (p ≤ 0.05).

### Exometabolomic analysis

Four-week-old Arabidopsis plants were sprayed with fine mist of either 10 mM MgCl_2_ mock solution or bacterial inoculum (2 x 10^8^ CFU.ml^-1^, in 10mM MgCl_2_). Each biological replicate consisted of five fully developed leaves from each plant and four replicates were sampled at 4 hours post inoculation (HPI) corresponding to ZT8 (ZT: zeitgeber time, where ZT0 refers to lights on). Leaf petioles were sealed with parafilm to prevent leaf fluid leakage, leaf blades were submerged into 5 ml of phosphate buffered saline, pH 7.4 (Genesee Scientific, El Cajon, CA, USA) and washed at 200 rpm for 1 hour. All 5 ml of the leaf washes were filtered through a 0.22-micron sterile membrane (Neta Scientific, Hainesport, NJ, USA) to remove bacterial cells and flash froze for downstream analysis. The absence of bacterial cells in the leaf wash samples was confirmed by serial dilutions and plating as previously described [74]. No bacterial cells were detected on the agar plates.

*Mass spectrometry analysis*: Frozen leaf wash samples were processed by the West Coast Metabolomics Center at the University of California, Davis (https://metabolomics.ucdavis.edu). The relative amounts of primary metabolites were quantified using gas chromatography-time of flight (GC-TOF) mass spectrometry as previously described by Fiehn et al. [75]. Briefly, leaf washes were dried down and resuspended with a mixed solution of retention index (RI) markers using fatty acid methyl esters of C8, C9, C10, C12, C14, C16, C18, C20, C22, C24, C26, C28 and C30 linear chain length. An Agilent 6890 gas chromatograph (Agilent, Santa Clara, CA) controlled by Leco ChromaTOF software version 2.32 was used to run the samples. This chromatograph system contained a 30-m long and 0.25-mm-internal-diameter Rtx-5Sil MS column with an additional 10-m long guard column. The sample injection volume was 0.5 µL and samples were introduced at -70 eV ionization energy with a temperature of 230 °C transfer line and 250 °C ion source. Raw data files were processed using ChromaTOF software and the resulting text files were exported to a data server with absolute spectra intensities. Metabolites were identified using a filtering algorithm implemented in the metabolomics PubChem database [76; https://pubchem.ncbi.nlm.nih.gov; accessed in August 2021]. Quantification was reported as peak heights at the specific retention index. The raw data were normalized according to procedures described by Fiehn [77], which consider the sum of all peak heights for all identified metabolites for each sample.

*Metabolomic data analysis*: The dataset containing the peak heights of all detected metabolites in all leaf wash samples (n = 4) were normalized by Log_10_ transformation and auto scaling (mean-centered and divided by the standard deviation of each variable) functions of the MetaboAnalyst software [78; https://www.metaboanalyst.ca/; accessed in December 2024]. To assess the quality of the data and the similarity among the sample profiles, the variation among replicates was evaluated using principal component analysis (PCA) and hierarchical clustering analysis using the MetaboAnalyst software. All metabolites were categorized based on their compound class by the R package omu 1.1 [79] using Kyoto Encyclopedia of Genes and Genomes (KEGG) IDs as an identifier (https://www.kegg.jp) and PubChem database.

To identify differentially accumulated metabolites (DAMs) among the three different treatments (*Pst* DC3000, STm 14028s, or mock) at 4 HPI, significant statistical differences among the means were calculated with ANOVA and Fisher’s least significant difference (LSD) post-hoc analysis. Significance was defined with the false discover rate (FDR p-value < 0.05) using the MetaboAnalyst software. The normalized peak heights of individual DAMs with a known classification were compared among all treatments using ANOVA and Tukey’s test (α = 0.05). The results from these comparisons were used to create the Venn diagrams and heatmaps.

*Data mining of previous studies*: Previously, Montano et al. [32] identified STm 14028s genomic regions that are required for bacterium-induced stomatal opening in lettuce. We searched these genomic regions for bacterial genes involved in the biosynthesis, regulation, or transport of DAMs identified in this study. In addition, Jacob et al. [33] reported dual transcriptomic analyses of Arabidopsis and STm 14028s. We searched their differential gene expression datasets for both Arabidopsis and STm 14028s genes that could be involved in the pathways associated with the identified DAMs.

### Creation of the O157:H7 (*ipdC*) Strain

STm 14028s was cultured overnight to an OD_600_ of 1.0 and DNA was extracted with the Zyppy Plasmid MiniPrep Kit (Zymo Research, Irvine, CA, USA). The STm 14028s indole pyruvate decarboxylase (*ipdC*) gene was amplified via PCR (GoTaq Green Master Mix, Promega, Madison, WI, USA) with specific primers (S1 Table) following the manufacturer’s protocol and confirmed via gel electrophoresis. The plasmid pET28a was isolated from an *E. coli* DH5α stock containing the plasmid via the Zyppy Plasmid Miniprep Kit and was linearized by cutting with the enzymes NdeI (NEB) and XhoI (NEB) according to the manufacturer instructions. The STm 14028s *ipdC* gene and linearized pET28a were assembled using Gibson Assembly Master Mix (NEB) according to the protocol provided by the manufacturer.

O157:H7 cells grown to an OD_600_ of 0.8 overnight were made competent using a method previously described [80]. Competent O157:H7 cells were transformed with the constructed pET28a plasmid containing STm 14028s *ipdC* via electroporation (Electroporator 2510, Eppendorf, Hamburg, Germany) with a single pulse of 2500 V and rescued by the addition of 1 ml SOC medium (20 g.L^-1^ tryptone, 5 g.L^-1^ yeast extract, 0.5 g.L^-1^ NaCl). Transformed cells were grown for one hour at 37 °C on an orbital shaker at 200 rpm and then plated on LSLB medium supplemented with 50 μg.ml^-1^ streptomycin and 50 μg.ml^-1^ kanamycin. Colonies were picked with a sterile toothpick, homogenized in 20 µL sterile water and heated at 80 °C for 10 minutes. A PCR (GoTaq Green Master Mix) reaction was carried out with 0.4 µM forward and reverse primers (S1 Table) following the manufacturer’s guidelines. Colony PCR results were compared to the wildtype STm 14028s by gel electrophoresis to select for positive O157:H7 (*ipdC*) colonies.

### Indole-3-acetic acid (IAA) Quantification

To test whether the strains could produce IAA, we cultured the cells in LSLB as described above and diluted the culture to an OD_600_ of 0.2 in 5 mL of M9 minimal medium [1X M9 salts (64 g.l^-1^ Na_2_HPO_4_•7H_2_O, 15 g.l^-1^ KH_2_PO_4_, 2.5 g.l^-1^ NaCl, 5.0 g.l^-1^ NH_4_Cl), 2 mM MgSO_4_, 100 µM CaCl_2_, 0.4% glucose] supplemented with 1 mM L-tryptophan (Sigma-Aldrich, MO, USA) as recommended by Cox et al. [22]. Cell cultures and a blank solution were incubated at 28 °C and 150 rpm for 72 hours. Bacterial cells were removed through 0.22-micron syringe filters (Thermo Fisher Scientific) and the liquid was flash frozen in liquid nitrogen.

Production of IAA was also assessed when bacterium was exposed to plants. Four-week-old wildtype Arabidopsis plants were sprayed with a fine mist of mock solution (10 µM MgCl_2_) or bacterial inoculum (5 x 10^8^ CFU.ml^-1^). At 4 HPI, five leaves were taken from the middle of the inoculated rosette and carefully placed in a 50 mL tube with 5 mL of 1X phosphate saline buffer (PBS; Thermo Fisher Scientific). The PBS solution was only in contact with the intact leaf blade surface to avoid contamination with exudates from the cut petiole. Samples were incubated at 28 °C and 250 rpm for 1 hour. Leaves were removed and the liquid was filtered through a 0.22-micron sterile membrane (Neta Scientific, Hainesport, NJ, USA) before being flash frozen in liquid nitrogen.

All frozen samples from the experiments above were concentrated by lyophilization overnight and resuspended in 250 µL of sterile water for the quantification of IAA production. IAA levels of collected samples were measured using an ELISA kit (Catalog #: MBS269958, MyBioSource, San Diego, CA, USA) following the manufacturer’s instructions and the OD_450_ was measured with a plate reader (Synergy H1, BioTek Instruments, Inc, Winooski, VT, USA). IAA concentrations were determined by comparison to the standard curve created with chemicals supplied by the ELISA kit. Data points represent the mean of three biological replicates (with two technical replicates each) ± SE. Statistical differences among the IAA levels were determined by an ANOVA followed by a Scott Knott test to compare the means (α = 0.05).

### Transcriptomic data mining and gene expression validation

To gain insights on the regulation of the auxin signaling pathway in both Arabidopsis and STm 14028s that could be important for stomatal opening, we examined our dual transcriptomic analysis of Arabidopsis, lettuce, and STm 14028s reported by Jacob et al. [33]. We searched for their differential gene expression datasets for both STm 14028s and Arabidopsis genes annotated as being involved in the pathways associated with auxin and ABA. Relevant differentially expressed genes (DEGs) in STm 14028s and Arabidopsis are listed in S3 Dataset and S4 Dataset, respectively.

Four genes involved in Arabidopsis auxin biosynthesis were selected for further validation by reverse transcriptase-quantitative PCR (RT-qPCR). Three leaves of vacuum-infiltrated plants, a procedure that inoculate both the leaf surface and apoplast, were collected from each treatment, including mock (0.004% Silwet L-77, PhytoTech Labs, Lenexa, KS, USA) or 1 x 10^8^ CFU ml^-1^ of each bacterium strain (STm 14028s, STm 14028s ∆*ipdC*, O157:H7, O157:H7 (*ipdC*), and *Pst* DC3000). At 4 HPI, leaf tissue was mechanically disrupted using a tissue grinder (Mix Mill MM 400, Retsch, Haan, Germany) twice for 1 minute at 30 Hz; the tissue was kept frozen throughout using liquid nitrogen. Total RNA was extracted using the RNeasy Plant Mini Kit (QIAGEN) according to the manufacturer’s instructions. RNA (800 ng) was reverse transcribed to cDNA using the Takara RNA PCR kit (Clontech, Mountain View, CA, USA) according to the manufacturer’s guidelines. cDNA was used for the RT-qPCR reaction using Fast SYBR™ Green Master Mix (Thermo Fisher Scientific) in accordance with the manufacturer’s protocol and read with a BioRad CFX Connect Real-Time PCR Detection System. Primer sequences are listed in S1 Table.

The expression level of *CYP71A12*, *NIT2*, *YUC5,* and *YUC6* are relative to reference gene *PP2AA3* (At1G13320) based on ΔCq values and further normalized to their expression level in mock-treated samples using the formula 2^-ΔΔCq^ [81]. Data points are the mean of four biological replicates with two technical replicates ± SE. Statistical differences among treatments were determined by an ANOVA and a comparison of the means was conducted by a Scott Knott test (α = 0.05).

### Accession numbers

Col-0 and mutant plant seeds were obtained from the Arabidopsis Resource Center (ABRC). Arabidopsis Genome Initiative identification numbers for the gene discussed in the article are as follows: *PP2AA3* (At1g13320, RT-qPCR reference gene), *CYP71A12* (AT2G30750), *NIT2* (AT3G44300), *YUC5* (At5g43890), *YUC6* (At5g25620), *AXR1* (At1g05180), *AUX1* (At2g38120), and *TIR1* (At3g62980).

The genome sequences of STm 14028s, O157:H7, and *Pst* DC3000 are available at the DNA DataBank of Japan (DDBJ), the European Nucleotide Archive (ENA), and GenBank at NBCI under the accession numbers JBCEWJ000000000, JBAGKT000000000 and JARFJH000000000, respectively.

Metabolomic data was deposited onto the EMBL-EBI MetaboLights database [82] with the identifier number MTBLS9884.

## Supporting information

S1 Dataset All primary metabolites detected in leaf washes of the Arabidopsis ecotype Col-0 when inoculated with Pst DC3000, STm 14028s, or mock solution. Leaf wash was sampled in four biological replicates at 4 hours post inoculation. Average peak heights of each metabolite and the value of individual biological replicates (BR) are shown in different columns. The statistical significance was determined using ANOVA and Fisher’s LSD post-hoc analysis and the significance was defined with the false discover rate (FDR) p-value < 0.05. All significantly accumulated metabolites are highlighted in red. Classification was assigned based on PubChem database with respective KEGG identification number using the R package omu 1.1 [79].
(XLSX)

S2 Dataset All 29 differentially accumulated metabolites (DAMs). The peak heights were normalized with the Log10 transformation and auto scaling (mean-centered and divided by the standard deviation of each variable) functions of the MetaboAnalyst software. The average of normalized peak heights of each metabolite and individual biological replicates (BR) are shown in different columns. The statistical significance was determined using ANOVA followed by Tukey’s test (α = 0.05). Classification was assigned based on respective KEGG identification number using the R package omu 1.1 [79] and PubChem database (pubchem.ncbi.nlm.nih.gov).
(XLSX)

S3 Dataset List of significantly up-regulated genes in STm 14028s at 4 HPI on both Arabidopsis and lettuce [data mined from 33]. Significantly differentially expressed genes were considered based on a Benjanimi–Hochberg false discovery rate adjusted p-value (adj.P.Value) <0.05 and a Log2 fold change (logFC) <-1 or >1.
(XLSX)

S4 Dataset Auxin- and ABA-related genes that are modulated by STm 14028s in Arabidopsis based on the transcriptomic analysis reported by Jacob et al. [33]. Genes are grouped by KEGG pathway classification. Significantly differentially expressed genes were considered based on a Benjanimi–Hochberg false discovery rate adjusted p-value (adj.P.Value) <0.05 and a Log2 fold change (logFC) <-1 or >1. Blue and red cells indicate significantly down- and up-regulated genes, respectively.
(XLSX)

S1 Table Sequences of primers used for RT-qPCR, plant genotyping, and cloning. (DOCX)

S1 Fig Metabolomic profile of leaf washes. (A) A workflow diagram of the experimental design for the leaf wash sample collection and analyses, repeated with four biological replicates. Created in BioRender.com. (B, C) Exometabolomic profile of metabolites in exudates of leaves exposed to different treatments. The peak height of all 438 metabolites detected at 4 hours post-inoculation (hpi) with mock, Pst DC3000, or STm 14028s were normalized by Log_10_ transformation and auto scaling (mean-centered and divided by the standard deviation of each variable) functions of the MetaboAnalyst software and used as input for the principal component analysis (PCA) (B) and hierarchical clustering analysis of all detected metabolites (C).
(TIF)

S2 Fig Confirmation of *ipdC* transformation into O157:H7 cells. Agarose gel showing the fragments amplified by colony PCR using primers IpdC_pET28a F and IpdC_pET28a R (S1 Table). The first lane shows the DNA molecular weight maker and respective sizes in kilobases (kb). The second to forth lanes show DNA fragments from colonies of STm 14028s, STm 14028s ∆*ipdC*, untransformed O157:H7, and O157:H7 (*ipdC*).
(TIF)

S3 Fig Modulation of Arabidopsis auxin pathway genes upon STm 14028s inoculation. A simplified version of the auxin pathway starting from the tryptophan (Trp) precursor, anthranilate. Arrows represent enzymatic steps between metabolites. Green boxes are representative of protein families involved and gray boxes represent biological processes. Adjacent to the steps of the pathway is the expression data [33] for the genes that encode the enzymes involved in the pathway. The data points are the average Log2 fold change (FC) in gene expression in Arabidopsis Col-0 vacuum-infiltrated with a mock solution or STm 14028s inoculum (1 x 10^9^ CFU.ml^-1^) at 4 HPI (n = 3). Significantly differentially expressed genes were considered genes with a Benjanimi–Hochberg false discovery rate adjusted p-value < 0.05 where ** = adjusted p-value of 0.01 and 0.001, and *** = adjusted p-value < 0.001. The yellow star indicates the genes tested by RT-qPCR.
(PDF)

S4 Fig Arabidopsis auxin mutant genotyping. (A) Representative images of Arabidopsis plant mutants and wildtype used in this screening. (B-F) Genomic DNA for each mutant plant and wildtype was used in PCR amplification with primers found on S1 Table. (B, C) The genotyping of *aux1-7* (CS3074), including the (B) PCR product of *aux1-7* and wildtype and (C) the results of a restriction enzyme (RE) digest of the PCR product with DpnII. The wildtype had two cut sites while *aux1-7* had three. (D-E) The genotyping of *axr1-3* (CS3075), including (D) PCR product of *axr1-3* and wildtype and (E) the results of a RE digest of the PCR product with AccI, which only cuts the wildtype allele. (F) Genotyping of *tir1-10* (Salk_090445C) with primers specific to the wildtype allele (LP + RP) and mutant allele (LBb1.3 + RP).
(TIF)

S5 Fig Morphological characteristics of stomata in Arabidopsis auxin mutants. (A) Diagram of the measurements taken from stomata, W = width of the complex, L = length of the complex, A = aperture width. Average measurements of the (B) stomatal aperture width, (C) width of the stomatal complex, (D) length of the stomatal complex, (E) width of the guard cell pair (W-A), (F) size of the stomatal complex (W*L). The same stomata were used to obtain the measurements. Results are shown as the mean of three independent experiments (n = 100) ± standard error (SE). (G) The stomatal density on the abaxial leaf surface. Results are displayed as the average number of stomata per mm^2^ (n = 20) ± SE. Statistical significance between the Col-0 and each of the mutants was detected by a two-tailed Student’s t-test (p ≤ 0.05; n.s. = non-significant).
(TIF)
