# Supplementary material for: Salmonella enterica exploits the auxin signaling pathway to overcome stomatal immunity
Source: PLoS Pathog. 2025 Nov 17;21(11):e1013662. doi: 10.1371/journal.ppat.1013662 (PMC12622826; doi:10.1371/journal.ppat.1013662)
Supplement: S1 Table — (DOCX) [file ppat.1013662.s005.docx]

| **Primer Name** | **Forward Sequence** | **Reverse Sequence** |
| --- | --- | --- |
| *RT-qPCR of Arabidopsis Genes* | | |
| *CYP71A12* | CTCTCCCAAGCGATGTTACGA | AGCATTGAAACCGTTGATCCT |
| *NIT2* | ATCGGAGCTGGTTGTGTTCC | ATTGTTCTTCCCGGCCAACT |
| *YUC5* | ACAAGTGGTCTCTGGCGCAT | TTCTCCGCGTTTTCTCCCGT |
| *YUC6* | TCGGTGCTCAGCCTTCTCTC | AACGGTCAACAAGCCGGATG |
| *PP2AA3* | CATGTTCCAAACTCTTACCTG | GTTCTCCACAACCGCTTGGT |
| *Arabidopsis Mutant Genotyping* | | |
| SALK_090445  (*tir1-10*) | CACGTGTCATCATCAGAATCG | ATTTCCCACCTCAGGAGATTC |
| CS3075  (*axr1-3*) | AAACCAACTTAACGTTTGCATGTCG | TCTCATATGTACTTTTCCTCGTCCTCTTCAC |
| CS3074  (*aux1-7*) | CATGGGTCAACAAAGCTTTGGATTTTGTCC | TTCGTGACTTTTACTCCCTTCACGTATACG |
| *Cloning of STm 14028s ipdC into pET28a* | | |
| IpdC_pET28a | GGTGGTGGTGGTGGTGCTCGAGCCAACCGTCACGAACCCT | GTGCCGCGCGGCAGCCATATGTTCCAGGCTTTCATCCCCA |

**Table S1.** Sequences of primers used for RT-qPCR, plant genotyping, and cloning.
