# Supplementary figures and images for: Salmonella enterica exploits the auxin signaling pathway to overcome stomatal immunity
Source: PLoS Pathog. 2025 Nov 17;21(11):e1013662. doi: 10.1371/journal.ppat.1013662 (PMC12622826; doi:10.1371/journal.ppat.1013662)

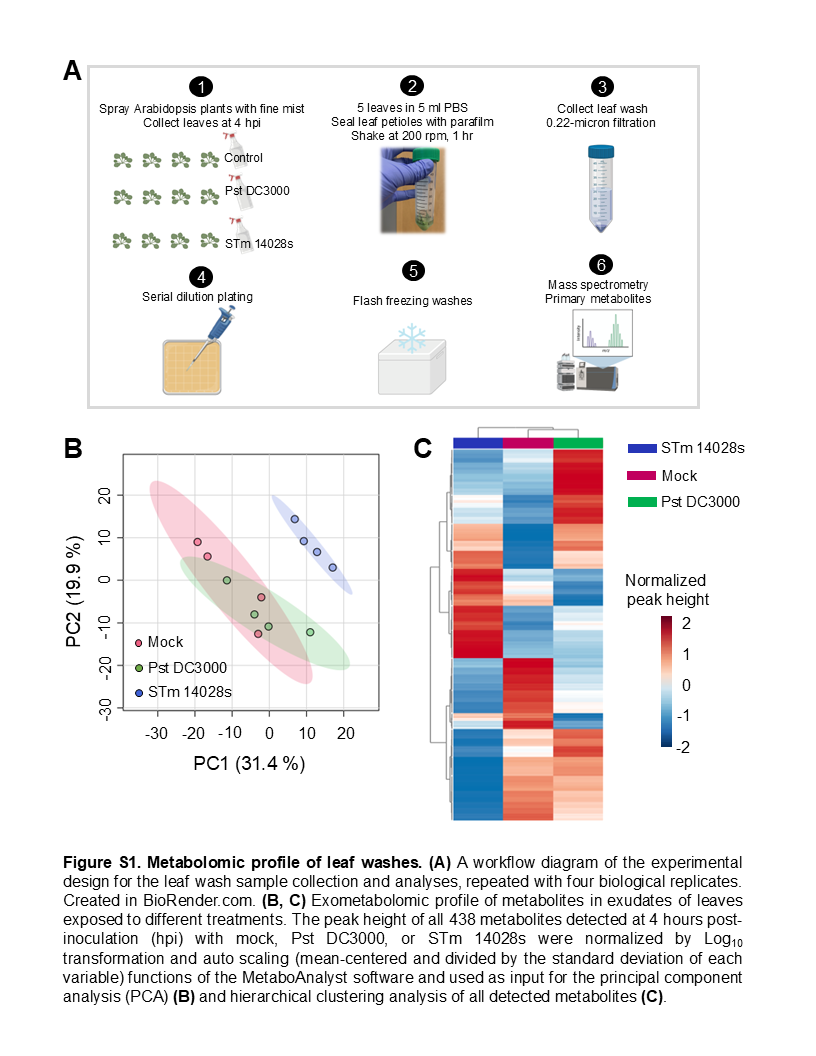

Supplement: S1 Fig — (A) A workflow diagram of the experimental design for the leaf wash sample collection and analyses, repeated with four biological replicates. Created in BioRender.com. (B, C) Exometabolomic profile of metabolites in exudates of leaves exposed to different treatments. The peak height of all 438 metabolites detected at 4 hours post-inoculation (hpi) with mock, Pst DC3000, or STm 14028s were normalized by Log10 transformation and auto scaling (mean-centered and divided by the standard deviation of each variable) functions of the MetaboAnalyst software and used as input for the principal component analysis (PCA) (B) and hierarchical clustering analysis of all detected metabolites (C). (TIF) [file ppat.1013662.s006.TIF]

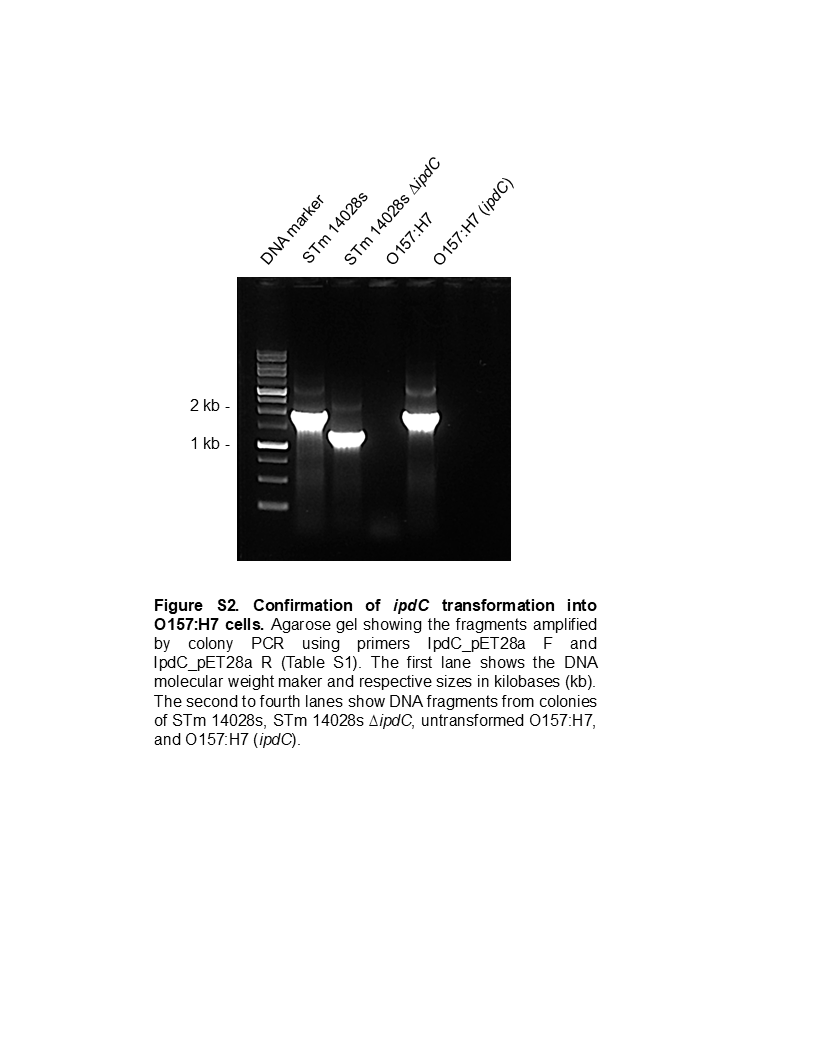

Supplement: S2 Fig — Agarose gel showing the fragments amplified by colony PCR using primers IpdC_pET28a F and IpdC_pET28a R (S1 Table). The first lane shows the DNA molecular weight maker and respective sizes in kilobases (kb). The second to forth lanes show DNA fragments from colonies of STm 14028s, STm 14028s ∆ipdC, untransformed O157:H7, and O157:H7 (ipdC). (TIF) [file ppat.1013662.s007.TIF]

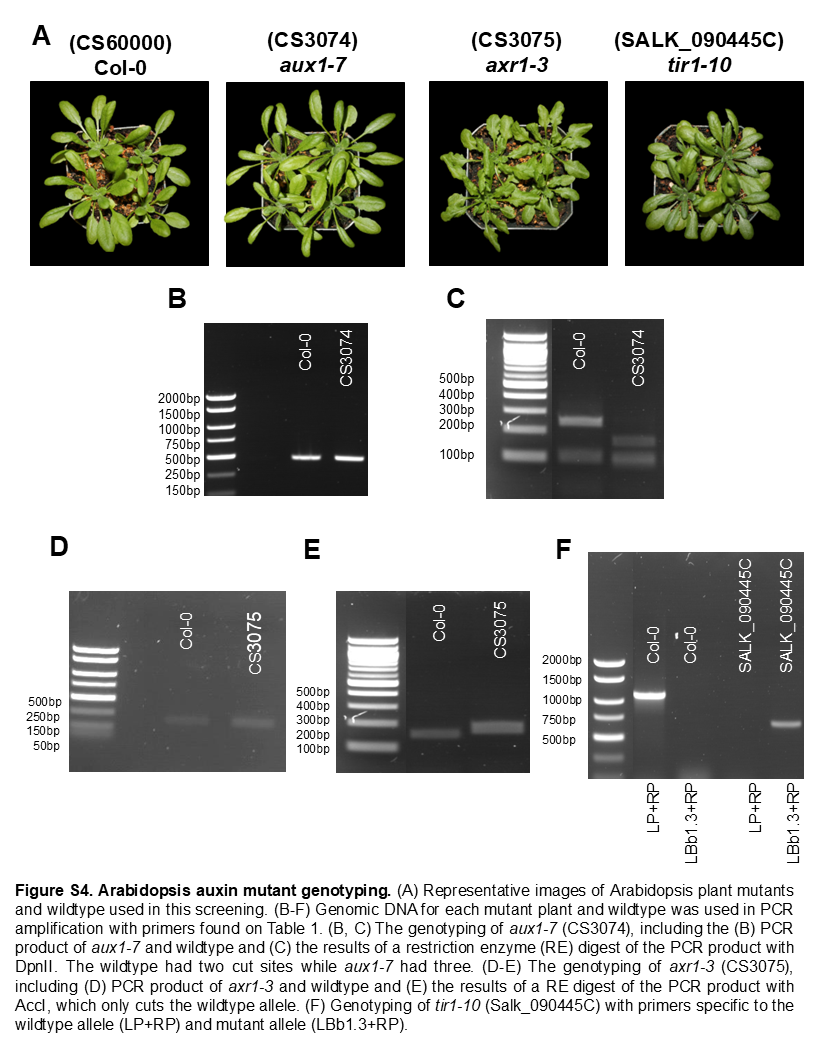

Supplement: S4 Fig — (A) Representative images of Arabidopsis plant mutants and wildtype used in this screening. (B-F) Genomic DNA for each mutant plant and wildtype was used in PCR amplification with primers found on S1 Table. (B, C) The genotyping of aux1-7 (CS3074), including the (B) PCR product of aux1-7 and wildtype and (C) the results of a restriction enzyme (RE) digest of the PCR product with DpnII. The wildtype had two cut sites while aux1-7 had three. (D-E) The genotyping of axr1-3 (CS3075), including (D) PCR product of axr1-3 and wildtype and (E) the results of a RE digest of the PCR product with AccI, which only cuts the wildtype allele. (F) Genotyping of tir1-10 (Salk_090445C) with primers specific to the wildtype allele (LP + RP) and mutant allele (LBb1.3 + RP). (TIF) [file ppat.1013662.s009.TIF]

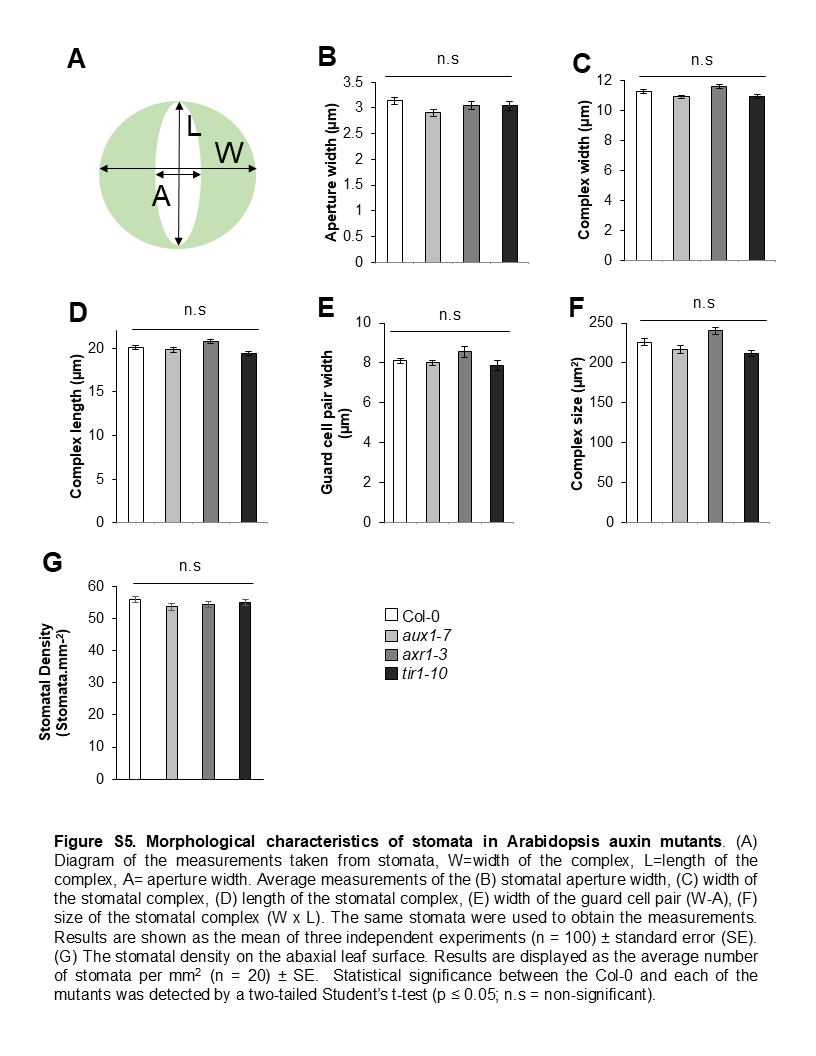

Supplement: S5 Fig — (A) Diagram of the measurements taken from stomata, W = width of the complex, L = length of the complex, A = aperture width. Average measurements of the (B) stomatal aperture width, (C) width of the stomatal complex, (D) length of the stomatal complex, (E) width of the guard cell pair (W-A), (F) size of the stomatal complex (W*L). The same stomata were used to obtain the measurements. Results are shown as the mean of three independent experiments (n = 100) ± standard error (SE). (G) The stomatal density on the abaxial leaf surface. Results are displayed as the average number of stomata per mm2 (n = 20) ± SE. Statistical significance between the Col-0 and each of the mutants was detected by a two-tailed Student’s t-test (p ≤ 0.05; n.s. = non-significant). (TIF) [file ppat.1013662.s010.TIF]
