# Supplementary material for: Salmonella enterica exploits the auxin signaling pathway to overcome stomatal immunity
Source: PLoS Pathog. 2025 Nov 17;21(11):e1013662. doi: 10.1371/journal.ppat.1013662 (PMC12622826; doi:10.1371/journal.ppat.1013662)
Supplement: S3 Fig — A simplified version of the auxin pathway starting from the tryptophan (Trp) precursor, anthranilate. Arrows represent enzymatic steps between metabolites. Green boxes are representative of protein families involved and gray boxes represent biological processes. Adjacent to the steps of the pathway is the expression data [33] for the genes that encode the enzymes involved in the pathway. The data points are the average Log2 fold change (FC) in gene expression in Arabidopsis Col-0 vacuum-infiltrated with a mock solution or STm 14028s inoculum (1 x 10^9^ CFU.ml-1) at 4 HPI (n = 3). Significantly differentially expressed genes were considered genes with a Benjanimi–Hochberg false discovery rate adjusted p-value < 0.05 where ** = adjusted p-value of 0.01 and 0.001, and *** = adjusted p-value < 0.001. The yellow star indicates the genes tested by RT-qPCR. (PDF) [file ppat.1013662.s008.pdf]

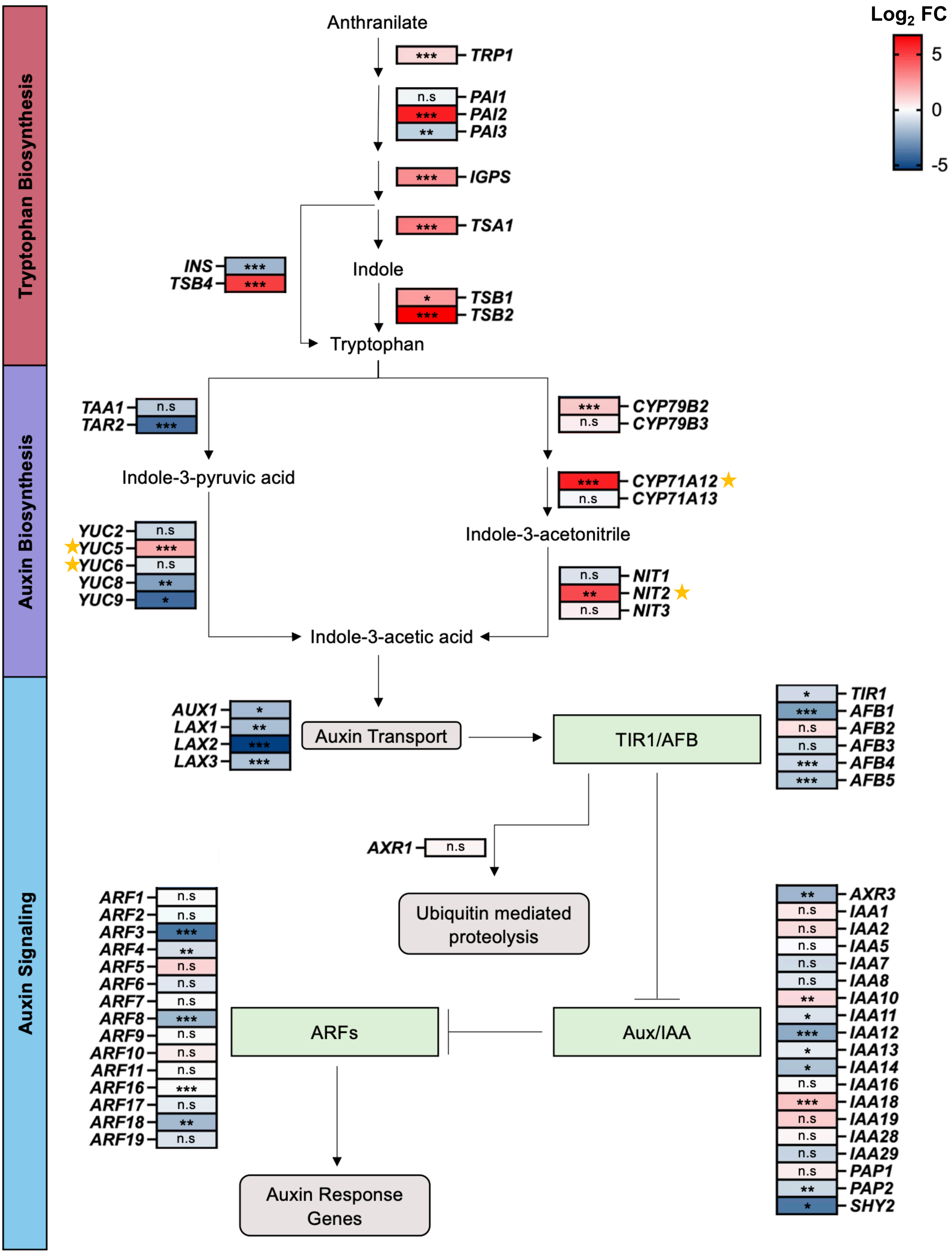

Tryptophan Biosynthesis
Auxin Biosynthesis
Auxin Signaling
Log2 FC
5
0
-5
Anthranilate
*** TRP1
n.s PAI1
*** PAI2
** PAI3
*** IGPS
*** TSA1
INS ***
TSB4 ***
Indole
* TSB1
*** TSB2
Tryptophan
TAA1 n.s
TAR2 ***
*** CYP79B2
n.s CYP79B3
Indole-3-pyruvic acid
*** CYP71A12
n.s CYP71A13
Indole-3-acetonitrile
YUC2 n.s
YUC5 ***
YUC6 n.s
YUC8 **
YUC9 *
n.s NIT1
** NIT2
n.s NIT3
Indole-3-acetic acid
AUX1 *
LAX1 **
LAX2 ***
LAX3 ***
Auxin Transport
TIR1/AFB
* TIR1
*** AFB1
n.s AFB2
n.s AFB3
*** AFB4
*** AFB5
AXR1 n.s
Ubiquitin mediated proteolysis
ARF1 n.s
ARF2 n.s
ARF3 ***
ARF4 **
ARF5 n.s
ARF6 n.s
ARF7 n.s
ARF8 ***
ARF9 n.s
ARF10 n.s
ARF11 n.s
ARF16 ***
ARF17 n.s
ARF18 **
ARF19 n.s
ARFs
Aux/IAA
** AXR3
n.s IAA1
n.s IAA2
n.s IAA5
n.s IAA7
n.s IAA8
** IAA10
* IAA11
*** IAA12
* IAA13
* IAA14
n.s IAA16
*** IAA18
n.s IAA19
n.s IAA28
n.s IAA29
n.s PAP1
** PAP2
* SHY2
Auxin Response Genes

Figure S3. Modulation of Arabidopsis auxin pathway genes upon STm 14028s inoculation. A simplified version of the auxin pathway starting from the tryptophan (Trp) precursor, anthranilate. Arrows represent enzymatic steps between metabolites. Green boxes are representative of protein families involved and gray boxes represent biological processes. Adjacent to the steps of the pathway is the expression data (Jacob et al., 2021) for the genes that encode the enzymes involved in the pathway. The data points are the average Log2 fold change (FC) in gene expression in Arabidopsis Col-0 vacuum-infiltrated with a mock solution or STm 14028s inoculum (1 x 10^9 $CFU.ml^{-1}$) at 4 HPI (n=3). Significantly differentially expressed genes were considered genes with a Benjanimi–Hochberg false discovery rate adjusted p-value < 0.05 where ** = adjusted p-value of 0.01 and 0.001, and *** = adjusted p-value < 0.001. The yellow star indicates the genes tested by RT-qPCR.
